# Effect of a Multispecies Synbiotic Supplementation on Body Composition, Antioxidant Status, and Gut Microbiomes in Overweight and Obese Subjects: A Randomized, Double-Blind, Placebo-Controlled Study

**DOI:** 10.3390/nu15081863

**Published:** 2023-04-13

**Authors:** Piyarat Oraphruek, Charoonsri Chusak, Sathaporn Ngamukote, Vorthon Sawaswong, Prangwalai Chanchaem, Sunchai Payungporn, Tanyawan Suantawee, Sirichai Adisakwattana

**Affiliations:** 1 Phytochemical and Functional Food Research Unit for Clinical Nutrition, Department of Nutrition and Dietetics, Faculty of Allied Health Sciences, Chulalongkorn University, Bangkok 10330, Thailand; 2 Center of Excellence in Systems Microbiology, Faculty of Medicine, Chulalongkorn University, Bangkok 10330, Thailand; 3 Department of Biochemistry, Faculty of Medicine, Chulalongkorn University, Bangkok 10330, Thailand

**Keywords:** probiotic, prebiotic, synbiotic, overweight and obesity, gut microbiota

## Abstract

Studies investigating the effect of multispecies synbiotic supplementation in obesity management are limited. The current study was performed to evaluate the effects of multispecies probiotics mixed with fructooligosaccharides on body composition, antioxidant status, and gut microbiome composition in overweight and obese individuals. We employed a randomized, double-blind, placebo-controlled trial design, in which 63 individuals aged 18–45 years were assigned to receive either a synbiotic supplement or placebo for 12 weeks. The synbiotic group consumed a daily dose of 37 × 10^9^ colony-forming units (CFU) of a unique blend of seven different probiotics, along with 2 g of fructooligosaccharides, while the placebo group consumed 2 g of maltodextrin daily. Assessments were performed at baseline, week 6, and the end of the study. The results of the study indicated that synbiotic supplementation resulted in a significant reduction in waist circumference and body fat percentage compared to the baseline measurements, as observed at 12 weeks. At the end of the study, there were no significant differences observed in body weight, BMI, waist circumference, or percentage of body fat between the synbiotic group and the placebo group. An analysis of plasma antioxidant capacity revealed that synbiotic supplementation caused a significant increase in Trolox equivalent antioxidant capacity (TEAC) and a concomitant decrease in malondialdehyde (MDA) in the test group when compared to the placebo. For the gut microbiota analysis, synbiotic supplementation significantly decreased *Firmicutes* abundance and the *Firmicutes*/*Bacteroidetes* (F/B) ratio at week 12 as compared to the placebo group. Nevertheless, the synbiotic group did not exhibit any substantial alterations in other biochemical blood parameters compared to the placebo group. These findings suggest that multispecies synbiotic supplementation could be a beneficial strategy to improve body composition, antioxidant status, and gut microbiome composition in overweight and obese subjects.

## 1. Introduction

Overweight and obesity can be attributed to a complex interplay of multiple factors that result in a dysregulated energy balance in the body, leading to an abnormal accumulation of adipose tissue or body fat. The prevalence of overweight and obesity has been recognized as a common epidemic and a crucial public health problem of the twenty-first century [1]. This is due to a combination of reduced physical activity and increased availability of high-calorie diets, which leads to an energy imbalance. One of the consequences of obesity is triggering a plethora of metabolic disturbances, including hypercholesterolemia, hyperglycemia, and hypertension, leading to diabetes and cardiovascular diseases (CVDs) [2,3]. The excessive deposition of adipose tissue in the body initiates a cascade of events that triggers the secretion of pro-inflammatory cytokines, namely tumor necrosis factor-α (TNF-α) and interleukin-6 (IL-6) [4]. These cytokines are known to play a key role in the development of the low-grade inflammation that characterizes obesity. In turn, this inflammation leads to the production of high levels of reactive oxygen species (ROS) and oxidative stress, which can cause damage to cellular components and contribute to the development of various diseases [4]. In addition, changes in the antioxidant capacity of the blood and an elevation in markers of inflammation have been associated with both obesity and metabolic syndrome [5,6]. Specifically, an imbalance in the gut microbiome characterized by an increase in the *Firmicutes/Bacteroides* (F/B) ratio has been shown to result in elevated levels of pro-inflammatory cytokines in obese subjects [7]. This altered proportion causes gut dysbiosis and impairs the ability to maintain intestinal homeostasis in the host [8]. 

Dietary interventions that include probiotics, prebiotics, or synbiotics (a combination of probiotics and prebiotics) have been discovered to restore the gut microbiota observed in obese individuals and promote weight loss and maintenance [9,10]. Recently, synbiotics may offer therapeutic benefits for overweight and obese individuals by modifying the gut microbiota composition, particularly in improving metabolic-syndrome-related parameters [11,12,13]. A systematic review also suggests that certain *Lactobacillus* species such as *L. gasseri*, *L. rhamnosus*, and *L. plantarum*, in combination with species from the *Bifidobacterium* genus, may help overweight and obese individuals lose weight and fat mass [14]. 

Despite some evidence that synbiotics may help reduce obesity and its associated consequences, research on the effect of synbiotics with multiple probiotic species in overweight and obese participants is limited. Therefore, the current study seeks to evaluate the impact of a specific multispecies synbiotic supplement, containing a blend of seven probiotic strains and fructooligosaccharides, on body composition, antioxidant levels, and gut microbiome composition in overweight and obese individuals. 

## 2. Materials and Methods

### 2.1. Participants

The study recruited participants from Chulalongkorn University in Bangkok, Thailand, through social media advertisements. Inclusion criteria for participants were: (1) being between the ages of 18 and 45, and (2) having a body mass index (BMI) between 23 and 30 kg/m^2^. The Asian BMI classification categorizes a BMI of 23–24.9 kg/m2 as ‘overweight’ and a BMI of 25–29.9 kg/m^2^ as ‘obese’. Therefore, individuals within this BMI range were considered overweight or obese, respectively, in our study. Participants with the following conditions were excluded from the study: (1) thyroid disorder or immune-compromised disease; (2) hepatitis; (3) cancer; (4) transplant patient; (5) having taken probiotic supplements within 2 weeks before screening; (6) having taken antibiotics within 4 weeks; (7) having received treatment for weight, energy expenditure, or glucose within the last 3 months; (8) having taken vitamin or mineral supplements within 6 months before screening; (9) being a current smoker; (10) being a current alcohol drinker; and (11) being pregnant or lactating. The study was approved by the Ethics Review Committee for Research Involving Human Research Subjects at Chulalongkorn University (COA No. 276/2563), and written informed consent was obtained from all participants prior to enrollment. The study protocol was retrospectively registered at Thaiclinicaltrials.org as TCTR20220828002.

### 2.2. Study Design

A double-blind, placebo-controlled, randomized, parallel design was conducted on overweight and obese individuals between March 2021 and January 2022 at Chulalongkorn University in Bangkok, Thailand. The total sample size required for this study was calculated to be *n* = 31, based on a significance level (α) of 0.05, 80% power, and the standard deviation of change (7.26) and mean change (5.15) in waist circumference from a previously described methodology [15]. We anticipated a 20% dropout rate and therefore aimed to recruit at least 74 participants for the study.

In the current study, a total of 80 individuals were enrolled for screening, but only 72 of them successfully completed the screening process. Participants were randomly assigned to either a placebo (*n* = 36) or synbiotic (*n* = 36) group based on their age, gender, body weight, and body mass index (BMI). The investigational product and placebo were sealed in sachets that were identical in appearance, labeling, and weight (2.03 g dry weight). The placebo consisted primarily of maltodextrins, and the synbiotic treatment (Lish flora^®^) contained 37 × 10^9^ CFU of multispecies probiotics (*Lactobacillus rhamnosus* LR3, *Lactobacillus gasseri* BNR17, *Lactobacillus salivarius* LS1, *Bifidobacterium lactis* BL2, *Bifidobacterium longum* BG3, *Bifidobacterium breve* BR2, *Bifidobacterium infantis* BT) mixed with fructooligosaccharides per sachet, provided by Absolute Well Being Co., Ltd., (Bangkok, Thailand). (Supplementary Table S1). To ensure double-blinding, the principal investigator, who was independent from both the data collection process and analysis, personally packed and prepared the assigned treatment for each participant. All participants were instructed to consume one sachet daily for 12 weeks before bedtime and to maintain their habitual diet and physical activity during the study. At baseline, in weeks 6 and 12, all experimental visits were preceded by an overnight fast (≥8 h) at Chulalongkorn University for anthropometrical measurements, blood collections, and dietary assessments. Participants were also instructed to avoid strenuous exercise for 24 h prior. Furthermore, fecal samples were collected at baseline and at the end of the study to analyze the change in gut microbiota composition. Product compliance was evaluated by counting the returned used and unused products at weeks 6 and 12. The consumed samples adhered to the protocol in more than 95% of the dispensed sachets.

### 2.3. Body Composition Assessment

Body weight, body mass index (BMI), and body fat percentage were assessed using bioelectrical impedance analysis (BIA) (TANITA BC-402, Tokyo, Japan) while participants were dressed in light clothing and no shoes [16]. Waist circumference was measured at the superior border of the iliac crest and the midpoint between the lowest rib and the iliac crest using a waist tape.

### 2.4. Biochemical Assessment

We collected venous blood samples using sodium fluoride and EDTA blood collection tubes for plasma samples and no anticoagulant tubes for serum samples. Blood samples were centrifuged at 3000 rpm for 15 min at 4 °C, and the plasma and serum were aliquoted and stored at −20 °C for further analysis. Plasma glucose, serum lipids, and kidney and liver function were analyzed by the Health Science Unit, Faculty of Allied Health Sciences, Chulalongkorn University, Thailand. Plasma antioxidants (TEAC) and plasma oxidative stress markers such as thiol and MDA (malonaldehyde) were evaluated at the phytochemical research unit, Chulalongkorn University, Thailand. 

The plasma Trolox equivalent antioxidant capacity (TEAC) was measured by the 2,2′-azinobis (3-ethylbenzothiazoline-6-sulfonate) free radical (ABTS●+) (Merck KGaA, Darmstadt, Germany) solution [17]. This reagent was prepared by a mixture of 7 mM ABTS in 0.1 M PBS (pH 7.4) and 2.45 mM K_2_S_2_O_8_ in distilled water (1:1 *v*/*v*) [18]. The ABTS●+ solution was incubated in the dark for 16 h. After that, the ABTS●+ solution was diluted with 0.1 M PBS (pH 7.4), and the absorbance was adjusted between 0.900 and 1.000 at 734 nm. Plasma TEAC was measured by incubating the adjusted ABTS^●+^ solution with plasma for 6 min and then measuring the reaction at 734 nm. The TEAC was expressed in μM Trolox (Sigma-Aldrich Co., St. Louis, MO, USA) equivalents.

The plasma protein thiol level was determined using a modified Ellman’s assay [19], in which a 1:10 diluted plasma sample (90 μL) was mixed with 2.5 mM 5,5′-dithiobis-(2-nitrobenzoic acid) (DTNB) (Merck KGaA, Darmstadt, Germany) in 0.1 M PBS (pH 7.4) and incubated at room temperature for 15 min. The reaction was then measured at 410 nm, and the plasma protein thiol level was expressed as mM L-cysteine (Sigma-Aldrich Co., St. Louis, MO, USA) equivalents.

The plasma MDA level was determined using a TBARS-based method [20]. A total of 200 μL of the plasma sample was mixed with 10% Trichloroacetic acid (TCA) (Merck KGaA, Darmstadt, Germany) and 50 mM 2,6-Di-tert-butyl-4-methylphenol (BHT) (Sigma-Aldrich Co., St. Louis, MO, USA) and then centrifuged at 12,000 rpm at 4 °C for 10 min. The liquid supernatant was mixed with 0.67% Thiobarbituric acid (TBA) (Sigma-Aldrich Co., St. Louis, MO, USA) and heated at 95 °C for 10 min. The absorbance was measured at 532 nm, and the plasma MDA level was expressed as μM Malondialdehyde tetrabutylammonium salt (MDA) (Sigma-Aldrich Co., St. Louis, MO, USA) equivalents.

### 2.5. Gut Microbiome Analysis

Stool samples were obtained from all participants, who were instructed to self-collect the specimens at home. Stools (100 g) from each participant were collected and stored at −80 °C in tubes containing DNA/RNA Shield™ solution (Zymo Research, Irvine, CA, USA). Participants were also provided with a poster describing the procedure and a video demonstrating how to collect the stool sample. All samples must be collected one to two days before the visit. The container was verified using the participant’s label number and sent for analysis of the 16S rDNA gene amplicon using polymerase chain reaction (PCR). DNA was extracted from stool samples using ZymoBIOMICS^TM^ DNA Miniprep Kit (Zymo Research, Irvine, CA, USA). Amplification of the full-length 16S rDNA gene was performed using specific primers and conditions as described previously [21]. The amplicons were barcoded using 5-cycle PCR using barcode primers based on the PCR Barcoding Expansion 1–96 (EXP-PBC096) kit (Oxford Nanopore Technologies, Oxford, UK). The PCR products were purified using QIAquick^®^ PCR Purification Kit (QIAGEN, Hilden, Germany). The concentration of purified PCR products was quantified by Qubit dsDNA HS Assay Kit using Qubit 4 fluorometer (Thermo Scientific, Waltham, MA, USA). The DNA libraries with different barcodes were pooled, end-repaired, adaptor-ligated, and then loaded onto the R10.4 flow cell for amplicon sequencing based on the MinlON Mk1C platform (Oxford Nanopore Technologies, Oxford, UK). Guppy basecaller software v6.0.7 [22] was applied for base calling with a super accuracy model. MinIONQC [23] was utilized to examine the quality of sequencing reads. Porechop v0.2.4 was used for demultiplexing and adaptor trimming. Reads were then clustered, polished, and taxonomically classified by NanoCLUST [24] using RDP database v11.5 [25]. The bacterial abundance and diversity analysis were demonstrated by Microbiome Analyst [26].

### 2.6. Dietary Assessment

At baseline and at weeks 6 and 12 of the study, participants were asked to complete a three-day dietary record (two weekdays and one weekend day) to estimate calorie intake. Additionally, all participants were asked to complete a 24 h dietary recall to ensure they maintained their intake and to reduce any bias from the food record. Nutrient intake was analyzed using the nutrient analysis software INMUCAL, Version 4.0 (Institute of Nutrition, Mahidol University, Nakhon Pathom, Thailand) [27]. 

### 2.7. Statistical Analysis

Data are represented as mean ± standard deviation (SD). The Kolmogorov–Smirnov test was applied to ensure a normal distribution of body composition, biochemical blood profiles, and gut microbiota composition. A paired sample *t*-test was performed to determine the difference between the results obtained at weeks 6 and 12 and the baseline, with *p* < 0.05 indicating statistical significance. To assess treatment group differences, an independent sample *t*-test was used at each time point to determine whether the treatment group’s results differed from the placebo group (*p* < 0.05). The Mann–Whitney U test was applied to compare alpha diversity analysis. Principal coordinate analysis (PCoA) was calculated based on Bray–Curtis distances, demonstrating sample clustering patterns. Permutational ANOVA (PERMANOVA) was used to compare the beta diversity using GraphPad Prism 6 software. The bacterial abundances were calculated at taxonomic levels of phylum and represented in relative abundance. A value of *p <* 0.05 was considered statistically significant. All statistical analyses were conducted using SPSS (version 16.0, SPSS Inc., Chicago, IL, USA).

## 3. Results

### 3.1. Participant Characteristics

**Figure 1:**
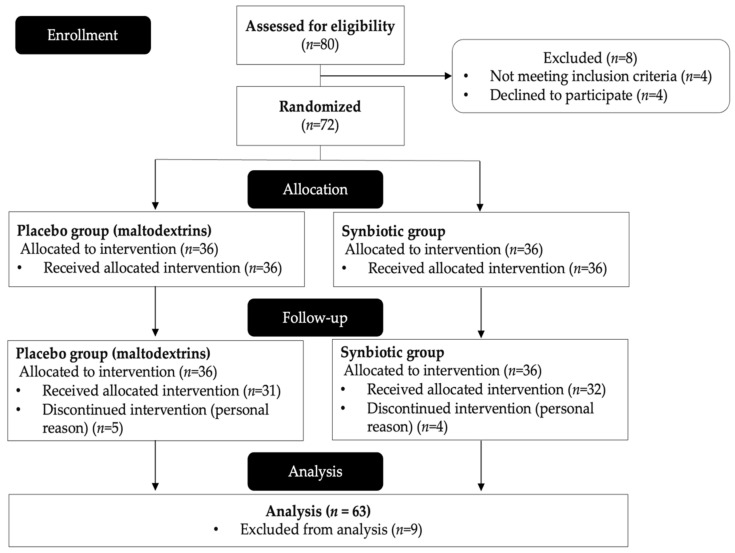
The CONSORT flow diagram of the study.

Eighty participants were recruited for the study (Figure 1). A total of 72 individuals were enrolled and randomized in the intervention. However, 9 participants (5 in the placebo group and 4 in the synbiotic group) discontinued and withdrew from the study due to personal reasons. As a result, only 63 participants (37 women, or 59% of the total, and 26 men, or 41% of the total) completed the 12-week study. At the beginning of the study, no significant differences were observed in age, height, body weight, BMI, waist circumference, or percentage of body fat between groups, as shown in Table 1.

### 3.2. Body Composition Measurement

The consumption of synbiotics for 12 weeks resulted in a significant decrease in waist circumference (*p* = 0.004) and body fat (*p* = 0.033) when compared to baseline measurements (Table 2). However, the synbiotics did not show significant differences in body weight or BMI reduction at weeks 6 and 12. Additionally, when compared to the placebo group, there were no significant changes in body weight, BMI, waist circumference, or percentage of body fat at the end of the study.

### 3.3. Biochemical Blood Profiles

After the synbiotic supplementation at 6 and 12 weeks, we observed no significant changes in plasma glucose, total cholesterol, triglycerides, HDL, or LDL compared to the baseline (Table 3). Interestingly, no significant differences were found in these biochemical parameters between the synbiotic and placebo groups at the 12-week mark. Additionally, there were no changes in the BUN, creatinine, uric acid, AST, or ALT levels in either group at the baseline or the end of the study (Supplementary Table S2).

Table 4 demonstrates the effect of a synbiotic intervention on plasma antioxidant status in overweight and obese participants. The results revealed that the group receiving the synbiotic supplement had a significantly higher plasma TEAC level than the placebo group at week 12. It is worth noting that both groups demonstrated an increase in plasma thiol levels at the end of the study compared to baseline measurements. However, there were no significant differences in thiol level between the synbiotic and placebo groups at weeks 6 and 12. Specifically, the placebo group displayed a significant increase in plasma MDA levels at week 12 compared to the baseline level. In contrast, the synbiotic group had a remarkable decline in MDA levels, and their levels were significantly lower than the placebo group by the end of the study. 

### 3.4. Dietary Measurement

The results of the dietary assessment, conducted after subjects consumed synbiotics, are presented in Supplementary Table S3. A noteworthy decrease in carbohydrate intake was observed in both groups at the 12-week mark compared to their baseline measurements. After the study was completed, no significant variations were observed in the overall intake of energy, carbohydrates, protein, or fat in comparison to the baseline measurements in either group.

### 3.5. Gut Microbiota Measurement

**Figure 2:**
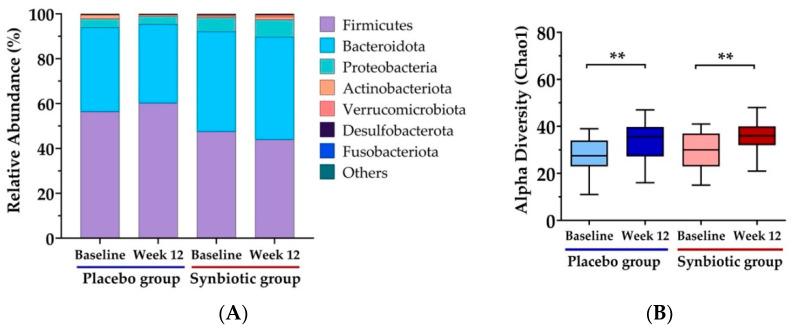
Effect of 12-week synbiotic intervention on gut microbiota composition in overweight and obese subjects. (**A**) Bar graphs show the phylum-level inter-individual variability in fecal microbiota. (**B**) The box plot shows the interquartile range, maximum value, and minimum value for the alpha diversity index: Chao1, which measures community richness. Values are represented as mean ± SD. ** *p*  <  0.01 compared to baseline.

The relative abundance of gut microbiome composition after synbiotic treatment is presented in Figure 2A. The most abundant gut microbiome compositions found in the feces of overweight and obese subjects were Firmicutes and Bacteroidota. Verrucomicrobiota, Proteobacteria, Patescibacteria, Fusobacteria, Desulfobacterota, Cyanobacteria, Campilobacterota, and Bdellovibrinota were also found in the feces. The alpha diversity index indicating community richness (Chao1) was used to evaluate the diversity of the gut microbiome. No significant differences in alpha diversity were observed between the groups at the start of the study. Both the synbiotic and placebo groups significantly increased alpha diversity at week 12 when compared with the baseline (*p* < 0.003 and *p* < 0.002, respectively), as depicted in Figure 2B.

**Figure 3:**
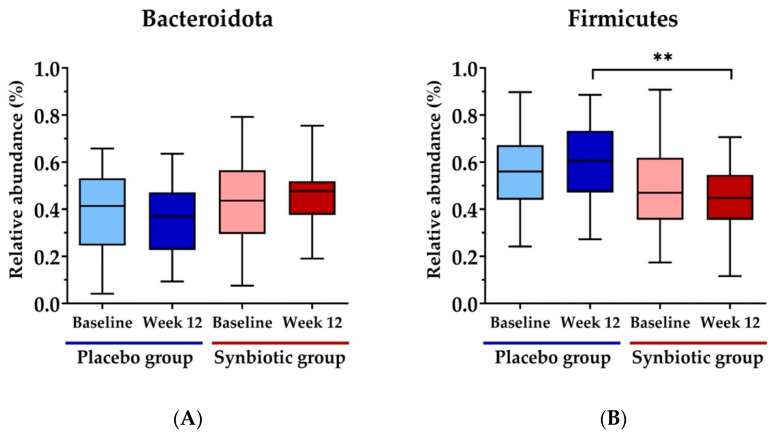
Percent relative abundance of dominant phylum across second treatment groups. (**A**) Phylum *Bacteroidota*, (**B**) phylum *Firmicutes*. Values are represented as mean ± SD. ** *p*  <  0.01 compared to placebo at week 12.

The percent relative abundance of the phyla Bacteroidota and Firmicutes did not differ at the baseline in either group (Figure 3A,B, respectively). Following the 12-week treatment, the abundance of Firmicutes at the phylum level exhibited a significant decrease in the synbiotic group compared to the placebo group (*p* < 0.003), as depicted in Figure 3B.

**Figure 4:**
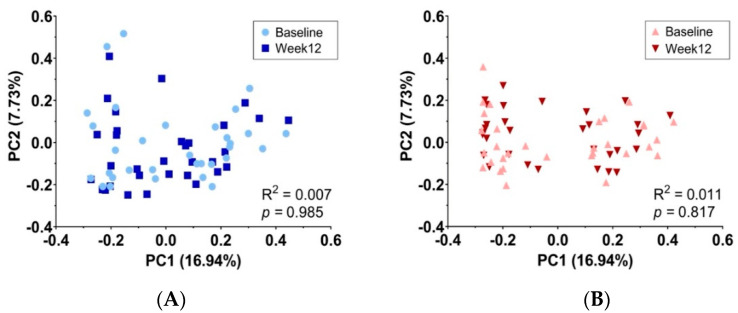

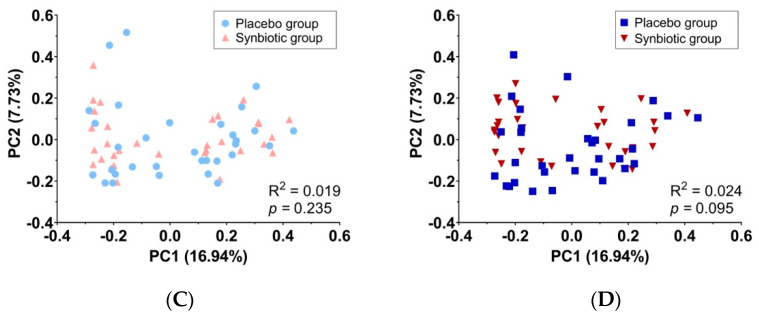
Principal coordinate analysis (PCoA) using Bray–Curtis distances demonstrates the beta diversity of the gut microbiome at the phylum level. Baseline vs. week 12 of (**A**) the placebo group and (**B**) the synbiotic group. The placebo group vs. the synbiotic group at (**C**) baseline and (**D**) week 12.

**Figure 5:**
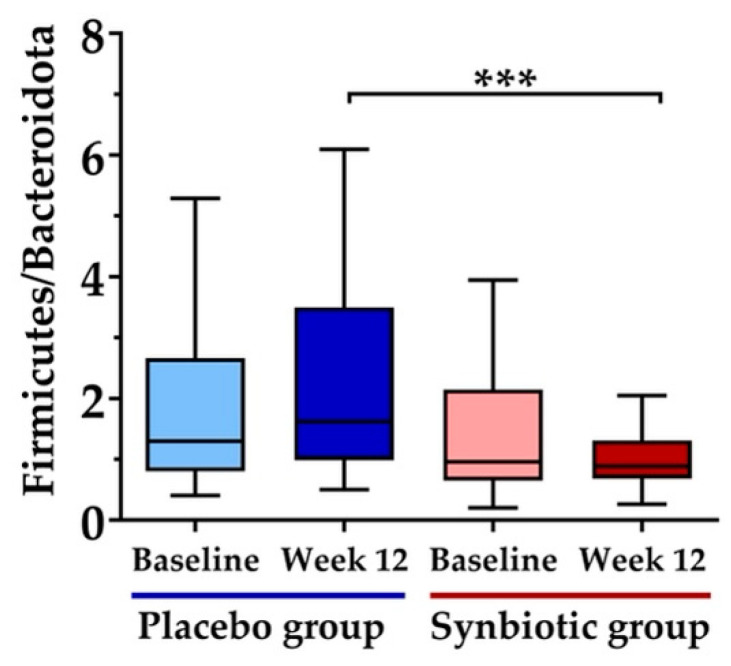
The effect of the 12-week synbiotic intervention on the *Firmicutes/Bacteroidota* (F/B) ratio in each experimental group was calculated using the relative abundance of bacteria. *** *p*  <  0.001 compared to the placebo group at week 12.

Figure 4 presents the principal coordinate analysis (PcoA) to demonstrate the beta diversity of the gut microbiome at the phylum level. No significant variations in beta diversity were observed at week 12 for either the placebo (Figure 4A) or synbiotic (Figure 4B) groups when compared to their baseline microbiome diversity. Additionally, there were no significant differences in beta diversity between the two groups at baseline (Figure 4C) and week 12 (Figure 4D) throughout the 12-week intervention. As demonstrated in Figure 5, the *Firmicutes*/*Bacteroidota* (F/B) ratio showed no significant difference within or between groups at the baseline. The consumption of synbiotics significantly decreased the F/B ratio at week 12 when compared with that of the placebo group.

## 4. Discussion

Recent research has indicated that synbiotic interventions may be a promising approach for overweight and obesity management [28,29]. Studies have demonstrated that synbiotics, a combination of probiotics and prebiotics, exhibit a more potent effect than probiotics or prebiotics alone, and that multi-strain probiotics are more effective and consistent than single-strain probiotics [30,31]. The literature recommends a minimum daily dosage of 106 CFU for multi-strain probiotics and 1–4 g of fructooligosaccharides (FOS) for prebiotics for a minimum of 10 weeks to effectively reduce gut dysbiosis and inflammation in patients with obesity and diabetes-related kidney disease [32]. Several studies have also supported the anti-obesity effects of certain bacterial strains, such as *Lactobacillus* spp. and *Bifidobacterium* spp., when mixed with prebiotics, by reducing BMI, waist circumference, and percentage body fat [33,34]. However, there is limited research on the effects of synbiotics on overweight and obesity management, which makes it challenging to establish conclusive evidence of their efficacy [35]. Therefore, to explore the effects of a multispecies synbiotic supplement on obesity, our current study is designed to assess the impact of long-term ingestion on body composition, antioxidant levels, and gut microbial composition in individuals who are overweight or obese.

The study highlights the potential benefits of a multispecies synbiotic intervention, combining probiotic bacteria (*Lactobacillus* and *Bifidobacterium*) and prebiotic components in managing obesity, with findings suggesting significant reductions in waist circumference and body fat percentage in overweight and obese subjects. The study participants who took the synbiotic for 12 weeks exhibited a highly significant decrease of 2.57% in their waist circumference compared to their baseline measurements, while a 0.92% increase in waist circumference was observed in the placebo group. Additionally, the synbiotic group also had a considerable reduction of 4.21% in their body fat percentage, while no significant changes were observed in the placebo group. These results suggest that multispecies synbiotic intervention may have a positive impact on body composition, particularly in terms of reducing the amount of visceral fat. The accumulation of visceral fat has been linked to an increased risk of non-communicable diseases (NCDs) [36]. Therefore, the observed reduction in waist circumference in the synbiotic group suggests that the intervention may positively impact reducing the amount of visceral fat in the body, which may in turn reduce the risk of these diseases. Our results align with previous studies, such as the investigations conducted by Rabiei et al., which have demonstrated reductions in waist circumference and body fat percentage through synbiotic supplementation containing comparable bacterial strains and prebiotics [37]. Minami et al. reported that the probiotic strain *Bifidobacterium breve* B-3 was effective in helping pre-obese adults reduce body fat. The study showed that after 12 weeks of treatment, the participants experienced a significant reduction in both body fat mass and percent body fat [38]. Moreover, a previous study has demonstrated the potential of *Lactobacillus gasseri* BNR17 in reducing waist circumference and visceral fat accumulation in obese subjects [39]. Based on the promising results of previous studies, it is reasonable to suggest that the inclusion of these probiotic strains in synbiotic interventions may have a positive impact on improving body composition in the current study. 

Changes in calorie intake can potentially impact alterations in waist circumference and body fat percentage among obese subjects. The findings showed that there were no significant differences in calorie intake, particularly in macronutrients, between the placebo and synbiotic groups. Therefore, decreased waist circumference and body fat percentage in the synbiotic group may not be directly attributed to changes in calorie intake. However, it is worth considering that the synbiotic intervention may have influenced the participants’ gut microbiome, leading to changes in energy metabolism and potentially promoting weight loss independent of calorie restriction.

Studies have shown that modulating the gut microbiota can increase energy expenditure and reduce fat storage in overweight and obese individuals [40]. The key aspect of the gut microbiota that has been the focus of much research is its richness, or the diversity and abundance of microbial species present in the gut. A growing body of evidence has linked gut microbiota richness to obesity, with studies showing that individuals who are overweight or obese tend to have a less diverse gut microbiome than those with healthy body weight [41,42]. In addition, a high diversity in gut microbes is associated with better health outcomes, while a lower diversity has been linked to several diseases, including obesity. Studies have shown that individuals with obesity tend to have a lower alpha diversity in gut microbiota, indicating a less diverse and balanced microbial community [43,44,45]. This imbalance in the gut microbiome is thought to contribute to the development of obesity by promoting inflammation, altering energy metabolism, and affecting appetite regulation. Given this evidence, using synbiotic supplementation to improve gut microbiota richness and diversity was hypothesized as a potential strategy for addressing obesity and related factors. Our study found that a 12-week intervention of synbiotic supplementation resulted in a significant increase in alpha diversity (Chao 1 index), indicating an increase in bacterial richness in the gut compared to the baseline. However, an increase in microbial diversity was found in the placebo group at the end of the study, suggesting that other factors such as race, genetics, diet, and lifestyle may have also played a role [46]. Beta diversity is normally used to highlight taxonomical gut microbiome differences between pairs of samples [47]. Our study found no significant differences in the community structure (beta diversity) at 12 weeks compared to the baseline, as determined by an analysis of distances within and between time points and groups. This finding is consistent with the findings of Sergeev et al. [48], who discovered that taking synbiotics containing *L. acidophilus*, *B. lactis*, *B. longum*, and *B. bifidum*, as well as a galactooligosaccharide mixture, did not result in a significant difference in beta diversity compared to a placebo after a 3-month intervention. The results of our study suggest that the synbiotic supplement may not have had a significant impact on the overall community structure of the gut microbiome, as evidenced by the lack of changes in beta diversity observed over the course of the 12-week intervention. There could be several possible reasons for this, such as the supplement lacking a sufficiently diverse range of bacterial strains, or the intervention being too brief to detect changes in beta diversity. Additionally, it is possible that the supplement is targeting specific microbial groups rather than promoting overall changes in the community composition.

The F/B ratio has been acknowledged to play a crucial role in maintaining intestinal homeostasis, and alterations in this ratio have been linked to various pathologies, including obesity [49]. Dysbiosis, characterized by an increase in the F/B ratio, has been associated with an increased abundance of *Firmicutes* and a decreased abundance of *Bacteroides* compared to healthy individuals [50,51]. *Lactobacillus* and *Bifidobacterium*, commonly used in treating gut dysbiosis in obesity, have been shown to restore the F/B ratio and reduce weight gain in overweight and obese individuals [52]. The F/B ratio did not differ at the start of the trial, but after a 12-week intervention, the synbiotic group showed a significant decrease in the ratio, while the control group had a slight increase. Several studies have found a link between synbiotic treatment, decreased F/B ratio, and reduced waist circumference and body fat [53,54,55]. These findings suggest that the multispecies synbiotic intervention may positively impact gut microbial health and improve body composition (waist circumference and percentage body fat) by reducing the relative abundance of *Firmicutes*.

Obesity-related metabolic disturbances are strongly associated with an elevation in oxidative stress, which refers to an imbalance between the body’s ability to repair or neutralize the damage caused by reactive oxygen species (ROS) and their production [56]. The excessive production of reactive oxygen species (ROS) can exceed the inherent antioxidant capacity of the body, resulting in oxidative damage to crucial macromolecules such as lipids, proteins, and DNA [57,58]. This leads to a decline in total antioxidant capacity (TAC) and thiol levels. The increase in oxidative stress has been strongly associated with the onset of various pathological conditions, including cardiovascular diseases, type 2 diabetes, and different forms of cancer, which can significantly elevate the risk of morbidity and mortality [59]. In the current study, the TEAC assay was used to measure antioxidant capacity in plasma, and it evaluated the plasma’s ability to scavenge the ABTS radical cation compared to Trolox. Our results showed no significant difference in plasma total antioxidant capacity (TEAC) at the beginning of the study in either the treatment or placebo groups. However, after consuming a synbiotic treatment for 12 weeks, there was a significant increase in TEAC in the synbiotic group when compared to baseline measurements. We employed the MDA assay as a marker for the assessment of lipid peroxidation. Our results demonstrated that the synbiotic intervention group had a significant decrease in MDA levels at the end of the study, while the placebo group exhibited a significant elevation in MDA levels when compared to their baseline measurements. These findings suggest that synbiotic treatment may positively impact antioxidant status and lipid peroxidation. Our findings are consistent with a previous study by Soleimani et al. which reported that consuming a synbiotic containing *L. acidophilus*, *L. casei*, and *B. bifidum* (2 × 10^9^ CFU/g each), along with 0.8 g/day of inulin, resulted in a significant increase in total antioxidant capacity, total glutathione levels, and a decrease in MDA levels after 12 weeks of treatment, compared to the placebo group [60]. While previous studies have not identified significant changes in antioxidant levels following the consumption of a specific synbiotic containing certain probiotic strains (2 × 10^10^ CFU of *L. paracasei*, 1 × 10^10^ CFU of *B. longum*, 2 × 10^10^ CFU of *B. breve*) and prebiotics (5 g of inulin and 5 g of FOS) for 12 weeks [61], our findings indicate that synbiotic consumption may have a beneficial impact on improving plasma antioxidant status. It is possible that the discrepancy in results may be attributed to the use of different probiotic strains in the intervention. Synbiotics have been shown to improve antioxidant activity, although the specific mechanisms behind this effect are not yet fully understood. One proposed explanation is that the production of short-chain fatty acids (SCFAs) by synbiotics may enhance glutathione synthesis, thereby improving antioxidant defenses [48]. Additionally, synbiotics may upregulate the expression of anti-inflammatory interleukin-18 and downregulate genes involved in oxidative stress and inflammation, further contributing to their antioxidant properties [62,63]. Recent studies have also investigated the relationship between the F/B ratio and inflammation [64]. The administration of *Lactobacillus acidophilus*, *Lactobacillus casei*, and *Bifidobacterium lactis* for 8 weeks in individuals with high BMI resulted in a reduction in body fat percentage and inflammation markers [65]. These findings suggest that synbiotics have the potential to modulate inflammation and improve multiple aspects of health.

The present study has several strengths, including a clinical approach, an appropriate sample size for the research question, a sufficient treatment period to detect changes in antioxidant status, interventions that align with the typical daily habits of participants, and no reported side effects from the synbiotic treatment. Moreover, recruiting overweight and obese participants in this study can provide valuable insights into understanding the impact of synbiotic interventions on this population and contribute to developing more effective strategies to address obesity. The study will be relevant to a high-risk population, and the results will be more generalizable to a broader range of patients. In this study, we aim to assess the efficacy of multispecies probiotics in overweight and obese individuals. This approach differs from previous studies that have utilized multi-strain probiotics. Using multispecies probiotics confers a more diverse range of beneficial bacterial strains and promotes a more diverse gut microbiome, which may enhance gut health and overall well-being. Additionally, multispecies probiotics have the potential to provide a more comprehensive range of health benefits compared to single-strain probiotics. However, the study also has some limitations that should be acknowledged. One limitation is the lack of fecal physical examination measurements, which could have provided valuable information about the effects of synbiotic consumption on gut health. Additionally, changes in the bacterial flora were not evaluated for specific bacterial strains, which could have provided insight into the impact of the synbiotic treatment on the F/B ratio. In order to fully comprehend the impact of synbiotics on antioxidant capacity, further studies should focus on evaluating the influence of individual variations and the microbiome composition on the efficacy of treatment.

## 5. Conclusions

The synbiotic treatment showed notable improvement in body composition (waist circumference and body fat percentage), antioxidant status, and gut microbiota (*Firmicutes* abundance and the F/B ratio) in overweight and obese individuals over 12 weeks. However, at the end of the study, there were no significant differences in body weight, BMI, waist circumference, or percentage of body fat between the synbiotic group and the placebo group. These findings suggest that synbiotic consumption may be a viable strategy for promoting overall health in these populations, although further research is warranted to determine its long-term effects.

## Figures and Tables

**Table 1:** Baseline characteristics of the participants.

Parameters	Placebo Group (*n* = 31)	Synbiotic Group (*n* = 32)	*p*-Value
Sex (male/female)	12/19	14/18	
Age (years)	33.69 ± 6.54	32.35 ± 6.53	0.422
Height (cm)	163.41 ± 10.71	163.74 ± 7.16	0.885
Weight (kg)	72.56 ± 13.22	73.86 ± 12.03	0.685
BMI (kg/m^2^)	27.00 ± 2.84	27.47 ± 3.30	0.547
Waist circumference (cm)	90.72 ± 10.37	91.01 ± 9.76	0.909
Percentage of body fat (%)	34.44 ± 8.45	35.85 ± 8.79	0.519

Values are represented as mean ± SD.

**Table 2:** Effect of 12-week synbiotic intervention on body composition in overweight and obese subjects.

Parameters	Baseline	Week 6	Week 12
Body weight (kg)			
Placebo group	73.86 ± 12.03 ^Aa^	73.93 ± 12.17 ^Aa^	73.82 ± 12.28 ^Aa^
Synbiotic group	72.56 ± 13.22 ^Aa^	72.55 ± 13.06 ^Aa^	72.10 ± 12.85 ^Aa^
Body mass index (kg/m^2^)			
Placebo group	27.47 ± 3.30 ^Aa^	27.50 ± 3.37 ^Aa^	27.46 ± 3.43 ^Aa^
Synbiotic group	27.00 ± 2.84 ^Aa^	27.01 ± 2.88 ^Aa^	26.85 ± 2.91 ^Aa^
Waist circumference (cm)			
Placebo group	91.01 ± 9.76 ^Aa^	90.25 ± 9.68 ^Aa^	91.85 ± 9.11 ^Aa^
Synbiotic group	90.72 ± 10.37 ^Aa^	89.18 ± 10.34 ^Ab^	88.39 ± 8.93 ^Ab^
Percentage of body fat (%)			
Placebo group	35.85 ± 8.79 ^Aa^	34.66 ± 8.14 ^Ab^	35.23 ± 8.62 ^Aa^
Synbiotic group	34.44 ± 8.45 ^Aa^	34.47 ± 8.11 ^Aa^	33.02 ± 8.26 ^Ab^

Values are represented as mean ± SD. Means in the same column with a different uppercase superscript (A: treatment effects) indicate a significant difference (*p* < 0.05) between groups. Means in the same row with a different lowercase superscript (a, b: time effect) indicate a significant difference (*p* < 0.05) when compared to baseline.

**Table 3:** Effect of 12-week synbiotic intervention on biochemical profiles in overweight and obese subjects.

Parameters	Baseline	Week 6	Week 12
Fasting plasma glucose (mg/dL)			
Placebo group	87.87 ± 7.54 ^Aa^	86.52 ± 8.08 ^Aa^	86.55 ± 8.24 ^Aa^
Synbiotic group	89.66 ± 9.39 ^Aa^	88.06 ± 10.03 ^Aa^	88.69 ± 11.73 ^Aa^
Total cholesterol (mg/dL)			
Placebo group	189.52 ± 42.78 ^Aa^	191.13 ± 38.30 ^Aa^	198.00 ± 34.71 ^Aa^
Synbiotic group	205.69 ± 31.64 ^Aa^	213.34 ± 41.26 ^Aa^	209.53 ± 34.24 ^Aa^
Triglyceride (mg/dL)			
Placebo group	90.10 ± 51.53 ^Aa^	119.16 ± 73.57 ^Ab^	123.61 ± 67.02 ^Ab^
Synbiotic group	91.72 ± 52.96 ^Aa^	107.75 ± 53.27 ^Ab^	113.94 ± 69.59 ^Ab^
HDL (mg/dL)			
Placebo group	49.81 ± 12.28 ^Aa^	49.68 ± 12.55 ^Aa^	51.58 ± 12.03 ^Aa^
Synbiotic group	51.16 ± 12.87 ^Aa^	50.16 ± 13.10 ^Aa^	50.66 ± 10.62 ^Aa^
LDL (mg/dL)			
Placebo group	126.13 ± 38.50 ^Aa^	127.55 ± 36.73 ^Aa^	122.84 ± 31.10 ^Aa^
Synbiotic group	141.03 ± 30.69 ^Aa^	153.38 ± 34.96 ^Bb^	135.84 ± 32.61 ^Aa^

Values are represented as mean ± SD. Means in the same column with a different uppercase superscript (A, B: treatment effects) indicate a significant difference (*p* < 0.05) between groups. Means in the same row with a different lowercase superscript (a, b: time effect) indicate a significant difference (*p* < 0.05) when compared to baseline.

**Table 4:** Effect of 12-week synbiotic intervention on plasma antioxidant status in overweight and obese subjects.

Parameters	Baseline	Week 6	Week 12
TEAC (µmol/L)			
Placebo group	1312.59 ± 155.59 ^Aa^	1303.81 ± 173.58 ^Aa^	1254.42 ± 195.54 ^Aa^
Synbiotic group	1265.07 ± 70.35 ^Aa^	1307.78 ± 170.22 ^Aa^	1340.34 ± 70.25 ^Bb^
Thiol (mmol/L)			
Placebo group	38.78 ± 8.46 ^Aa^	38.47 ± 6.07 ^Aa^	42.12 ± 5.73 ^Ab^
Synbiotic group	39.93 ± 5.50 ^Aa^	39.69 ± 3.92 ^Aa^	42.52 ± 5.05 ^Ab^
MDA (µmol/L)			
Placebo group	0.29 ± 0.31 ^Aa^	0.61 ± 0.47 ^Ab^	1.82 ± 0.94 ^Ab^
Synbiotic group	0.50 ± 0.44 ^Ba^	0.27 ± 0.49 ^Ba^	0.11 ± 0.11 ^Bb^

Values are represented as mean ± SD. Means in the same column with a different uppercase superscript (A, B: treatment effects) indicate a significant difference (*p* < 0.05) between groups. Means in the same row with a different lowercase superscript (a, b: time effect) indicate a significant difference (*p* < 0.05) when compared to baseline. TEAC; Trolox equivalent antioxidant capacity, MDA; malonaldehyde.

## Data Availability

The data presented in the manuscript are available on request from corresponding author.

## Supplementary Materials

The following supporting information can be downloaded at: https://www.mdpi.com/article/10.3390/nu15081863/s1.. Table S1: the compositions of synbiotic supplementation. Table S2: Effect of 12-week synbiotic intervention on kidney and liver function in overweight and obese subjects. Table S3: the three-day food record in placebo and synbiotic groups during the study.
