# Supplementary material for: Effect of a Multispecies Synbiotic Supplementation on Body Composition, Antioxidant Status, and Gut Microbiomes in Overweight and Obese Subjects: A Randomized, Double-Blind, Placebo-Controlled Study
Source: Nutrients. 2023 Apr 13;15(8):1863. doi: 10.3390/nu15081863 (PMC10141052; doi:10.3390/nu15081863)
Supplement: Supplementary file 1 [file nutrients-15-01863-s001.zip › nutrients-2278966-supplementary.pdf]

Table S1. The compositions of synbiotic supplementation.

Components	Contents	
	Placebo supplement	Synbiotic supplement
Maltodextrins	1365 mg	-
Fructooligosaccharides	-	995 mg
Lactobacillus rhamnosus LR3	-	10 mg
Lactobacillus gasseri BNR17	-	200 mg
Lactobacillus salivarius LS1	-	10 mg
Bifidobacterium lactis BL2	-	120 mg
Bifidobacterium longum BG3	-	10 mg
Bifidobacterium breve BR2	-	10 mg
Bifidobacterium infantis BT	-	10 mg
Flavors	465 mg	465 mg
INS296	100 mg	100 mg
Sucralose	100 mg	100 mg

Table S2. Effect of 12-week synbiotic intervention on kidney and liver function in overweight and obese subjects.

Parameters	Baseline	Week 12
Blood urea nitrogen; BUN (mg/dL)		
Placebo group	11.00 ± 3.04^{Aa}	12.16 ± 3.12^{Aa}
Synbiotic group	10.53 ± 2.66^{Aa}	12.06 ± 3.19^{Aa}
Creatinine (mg/dL)		
Placebo group	0.70 ± 0.19^{Aa}	0.72 ± 0.16^{Aa}
Synbiotic group	0.76 ± 0.19^{Aa}	0.75 ± 0.19^{Aa}
Uric acid (mg/dL)		
Placebo group	5.42 ± 1.48^{Aa}	5.82 ± 1.31^{Aa}
Synbiotic group	5.93 ± 1.40^{Aa}	6.38 ± 1.55^{Aa}
Aspartate aminotransferase; AST (U/L)		
Placebo group	18.35 ± 7.81^{Aa}	21.48 ± 12.87^{Aa}
Synbiotic group	26.75 ± 34.98^{Aa}	21.56 ± 10.12^{Aa}
Alanine aminotransferase; ALT (U/L)		
Placebo group	23.81 ± 20.39^{Aa}	28.19 ± 29.95^{Aa}
Synbiotic group	24.41 ± 16.89^{Aa}	26.63 ± 21.07^{Aa}

Values are represented as mean ± SD. Means in the same column with a different upper-case superscript (A-B: treatment effects) are a significant difference ($p < 0.05$). Means in the same row with a difference lower-case superscript (a-b: time effect) are a significant difference ($p < 0.05$) when compared with baseline.

Table S3. The three-day food record in placebo and synbiotics groups during the study.

Parameters	Baseline	Week 6	Week 12
Total energy (kcal/d)			
Placebo group	1448.30 ± 563.50^{Aa}	1401.26 ± 380.05^{Aa}	1150.94 ± 362.13^{Ab}
Synbiotic group	1499.38 ± 400.00^{Aa}	1339.86 ± 408.29^{Aa}	1316.22 ± 460.89^{Aa}
Carbohydrate (g/d)			
Placebo group	184.69 ± 72.95^{Aa}	164.84 ± 75.45^{Aa}	134.00 ± 50.25^{Ab}
Synbiotic group	186.07 ± 61.18^{Aa}	155.41 ± 48.44^{Ab}	155.79 ± 56.82^{Ab}
Protein (g/d)			
Placebo group	64.63 ± 32.41^{Aa}	42.47 ± 30.21^{Ab}	52.54 ± 16.51^{Aa}
Synbiotic group	63.84 ± 20.74^{Aa}	59.19 ± 23.66^{Ba}	58.49 ± 24.71^{Aa}
Fat (g/d)			
Placebo group	50.11 ± 27.45^{Aa}	54.58 ± 18.53^{Aa}	44.97 ± 18.98^{Aa}
Synbiotic group	55.53 ± 18.33^{Aa}	53.50 ± 22.97^{Aa}	51.01 ± 24.50^{Aa}

Values are represented as mean ± SD. Means in the same column with a different upper-case superscript (A-B: treatment effects) are a significant difference ($p < 0.05$). Means in the same row with a difference lower-case superscript (a-b: time effect) are a significant difference ($p < 0.05$) when compared with baseline.
